# Quantified Detection of Treponema pallidum DNA by PCR Assays in Urine and Plasma of Syphilis Patients

**DOI:** 10.1128/spectrum.01772-21

**Published:** 2022-03-22

**Authors:** Cuini Wang, Xin Zheng, Zhifang Guan, Danyang Zou, Xin Gu, Haikong Lu, Mei Shi, Pingyu Zhou

**Affiliations:** a STD Institute, Shanghai Skin Disease Hospital, Tongji University School of Medicine, Shanghai, People’s Republic of China; University of Mississippi Medical Center

**Keywords:** PCR, *Treponema pallidum* DNA, urine

## Abstract

Treponema pallidum can invade any organ, and T. pallidum DNA can be detected in various tissues and fluids. However, the knowledge of the presence and loads of T. pallidum DNA in urine is limited. For this study, we enrolled 208 syphilis patients (34 primary syphilis, 61 secondary syphilis, 68 latent syphilis, and 45 symptomatic neurosyphilis) and collected urine and plasma samples from them. *polA* and *Tpp47* genes were amplified in urine supernatant, urine sediment, and plasma using nested PCR and droplet digital PCR assays. The detection rates were 14.9% (31 of 208) and 24.2% (50 of 207) in urine supernatant and sediment, respectively (*P* = 0.017). The detection rates of T. pallidum DNA in urine sediment were 47.1, 47.5, 4.4, and 4.5% for primary, secondary, latent, and symptomatic neurosyphilis, respectively. After treatment, T. pallidum DNA in urine in 20 syphilis patients turned negative. Loads of T. pallidum DNA in urine sediment were significantly higher than those in plasma and urine supernatant (both *P* < 0.05). Our study indicated that T. pallidum DNA in urine could be found in patients at all stages of syphilis and showed high loads in urine sediment. Though it is unlikely to improve the routine diagnostic algorithm, the detection of T. pallidum DNA in urine may play certain roles in cases difficult to diagnose. In addition, urine is abundant and convenient to collect; therefore, urine sediment could be an ideal specimen for acquiring an amount of T. pallidum DNA that can be supplement samples for the detection of molecular typing of T. pallidum.

**IMPORTANCE** Syphilis is a sexually transmitted disease caused by Treponema pallidum sub. *pallidum.* T. pallidum can invade many organs, and T. pallidum DNA can be detected in various tissues and fluids. The results reported here demonstrated that T. pallidum DNA could be detected in urine in patients at all stages of syphilis. The detection rate and loads of T. pallidum DNA in urine sediment were significantly higher than those in urine supernatant. Urine is abundant, and its collection is noninvasive and convenient; therefore, urine is an ideal sample for acquiring a large amount of T. pallidum DNA, which can be supplement samples for the detection of molecular typing of T. pallidum.

## INTRODUCTION

The resurgence of syphilis is in rising globally (1). Known as a multistage sexually transmitted disease, the causative pathogen Treponema pallidum sub. *pallidum* (T. pallidum) can readily disseminate to many organs (2), and therefore T. pallidum DNA can be detected in various tissues and fluids by PCR assays (3–6).

Though the diagnosis of syphilis is mainly based on clinical manifestations combined with non-treponema and treponema serological tests, the detection of the nucleic acid of the pathogen from certain specimens is a direct method for the definite diagnosis of infectious diseases. According to previous studies, the detection rates of T. pallidum DNA in secondary syphilis patients reached 87.5 and 64.5% in saliva and oral swabs, respectively (4, 7). Blood T. pallidum DNA was positive in suspected primary syphilis patients whose blood rapid plasma regain (RPR) and T. pallidum particle agglutination (TPPA) were nonreactive (3). Recently, growing evidence showed that the nucleic acid of the pathogen in many infectious diseases was present in urine (8, 9). The detection of Borrelia burgdorferi DNA in urine was important in the laboratory diagnosis of Lyme borreliosis (10–12). Previously, two preliminary observations in a few samples indicated that T. pallidum DNA could be detected in urine (13, 14). Urine T. pallidum DNA was present in 6% of men who have sex with men (MSM) with early syphilis (15). In the present study, to comprehensively investigate the detection rate and loads of T. pallidum DNA in urine in syphilis patients at different stages, we enrolled 208 syphilis patients and detected T. pallidum DNA in urine and blood by nested PCR (nPCR) and droplet digital PCR (ddPCR) assays.

## RESULTS

### Clinical characteristics of the enrolled syphilis patients.

A total of 208 syphilis patients were enrolled, including 34 (16.3%) patients with primary syphilis, 61 (29.3%) with secondary syphilis, 68 (32.7%) with latent syphilis, and 45 (21.6%) with symptomatic neurosyphilis. As shown in Table 1, 21 patients (10.1%) were coinfected with HIV. All the patients were serum TPPA- and RPR-positive, except 4 possible primary syphilis patients with positive serum TPPA but negative RPR. All the patients with primary syphilis had genital chancres. Of 61 patients with secondary syphilis, 7 (11.5%) had periurethral lesions (1 patient with genital ulcer, 6 patients with condyloma latum). Among 45 symptomatic neurosyphilis, there were 7 (15.6%) patients with ocular neurosyphilis, 4 (8.9%) patients with meningovascular neurosyphilis, 33 (73.3%) patients with general paresis, and 1 (2.2%) patient with tabes and general paresis.

**TABLE 1:** Clinical characteristics of 208 syphilis patients enrolled in the study^*a*^

Variables	All cases (*n* = 208)	Primary (*n* = 34)	Secondary (*n* = 61)	Latent (*n* = 68)	Symptomatic neurosyphilis (*n* = 45)
Age, median (IQR) (yr)	51.5 (32.5 to 60)	39 (29 to 56)	36 (28 to 52)	55 (37 to 64)	58 (52 to 62)
Male, *n* (%)	134 (64.4)	33 (97.1)	32 (52.5)	29 (42.6)	40 (88.9)
Blood TPPA (+), *n* (%)	208 (100)	34 (100)	61 (100)	68 (100)	45 (100)
1/serum RPR titer (median, IQR)	32 (16 to 64)	16 (2 to 32)	64 (32 to 128)	32 (8 to 64)	64 (4 to 512)
Periurethral lesions, *n* (%)^*b*^	41 (19.7)	34 (100)	7 (11.5)	0 (0)	0 (0)
HIV infection, *n* (%)	21 (10.1)^*c*^	1 (3.0)^*d*^	19 (31.1)	0 (0)	1 (2.2)

*^a^*IQR, interquartile range; RPR, rapid plasma regain; TPPA, T. pallidum particle agglutination.

*^b^*“Periurethral lesions” refers to chancre and/or condyloma latum in the periurethral orifice.

c The total number of cases was 207 because the HIV test of 1 case was not done.

d The total number of cases was 33 because the HIV test of 1 case was not done.

### Sensitivity and specificity of *polA* and *Tpp47* detection in plasma and urine by nPCR assay.

To investigate the limit of detection (LOD) of *polA* and *Tpp47* in plasma and urine by nPCR assay, the different concentrations of T. pallidum from 3 × 10^4^ to 0 T. pallidum/mL were diluted with uninfected plasma and urine, and *polA* and *Tpp47* were then amplified by nPCR assay. As shown in Fig. S1, the LOD for *polA* and *Tpp47* genes in plasma were 3 and 15 T. pallidum/mL, respectively. The equal LOD (30 T. pallidum/mL) for *polA* and *Tpp47* in urine was observed. There were no amplification products when *polA* and *Tpp47* were amplified in control samples by nPCR assay.

### The detection rates of T. pallidum DNA in urine and plasma from syphilis patients.

*polA* and *Tpp47* were detected in plasma and urine from different stages of syphilis patients by nPCR assay. The samples were considered positive if *polA*, *Tpp47*, or both were positive. As shown in Table 2, the detection rates of T. pallidum DNA in urine supernatant and sediment were 14.9 and 24.2%, respectively, which was higher in urine sediment compared to that in urine supernatant (*P* = 0.017). The detection rate of T. pallidum DNA in plasma was 28.1% and was not significantly different from that in urine sediment (*P* = 0.37). The detection rates of T. pallidum DNA in urine sediment were 47.1, 47.5, 4.4, and 4.5% for primary, secondary, latent syphilis, and symptomatic neurosyphilis, respectively. The detection rates of T. pallidum DNA in urine sediment of patients with primary and secondary syphilis were significantly higher than in that in cases of syphilis and symptomatic neurosyphilis (all *P* < 0.05), and there was not significant difference between primary and secondary syphilis (*P* = 1.00). There was no significant difference between urine sediment and plasma at the same stage (all *P* > 0.05). In addition, T. pallidum DNA in urine supernatant and sediment from 20 syphilis patients after treatment were all undetectable.

**TABLE 2:** Detection rates of T. pallidum DNA in urine and plasma samples from syphilis patients by nested PCR (nPCR) assay

Stages	Urine supernatant			Urine sediment			Plasma		
	*polA*(+)	*Tpp47*(+)	*polA* and *Tpp47*(+)	*polA*(+)	*Tpp47*(+)	*polA* and *Tpp47*(+)	*polA*(+)	*Tpp47*(+)	*polA* and *Tpp47*(+)
Primary (*n* = 34)	23.5% (8 of 34)	26.5% (9 of 34)	26.5% (9 of 34)	47.1% (16 of 34)	41.2% (14 of 34)	47.1%^*f*^^,^^*g*^ (16 of 34)	40%^a^ (12 of 30)	36.7% (11 of 30)^*a*^	53.3%^a^ (16 of 30)
Secondary (*n* = 61)	23% (14 of 61)	18% (11 of 61)	31.1% (19 of 61)	45.9% (28 of 61)	42.6% (26 of 61)	47.5%^*f*^^,^^*g*^^,^^*h*^ (29 of 61)	50.8% (31 of 61)	47.5% (29 of 61)	60.7% (37 of 61)
Latent (*n* = 68)	1.5% (1 of 68)	2.9% (2 of 68)	2.9% (2 of 68)	4.4% (3 of 68)	2.9% (2 of 68)	4.4% (3 of 68)	1.5% (1 of 68)	2.9% (2 of 68)	2.9% (2 of 68)
Symptomatic neurosyphilis (*n* = 45)	0% (0 of 45)	2.2% (1 of 45)	2.2% (1 of 45)	0% (0 of 44)^*b*^	4.5% (2 of 44)^*b*^	4.5% (2 of 44)^*b*^	4.5% (2 of 44)^*c*^	2.3% (1 of 44)^*c*^	4.5% (2 of 44)^*c*^
All cases (*n* = 208)	11.1% (23 of 208)	11.1% (23 of 208)	14.9% (31 of 208)	22.7% (47 of 207)^*d*^	21.3% (44 of 207)^*d*^	24.2%^d^ (50 of 207)^*d*^^,^^*h*^	22.7% (46 of 203)^*e*^	21.2% (43 of 203)^*e*^	28.1% (57 of 203)^*e*^

a The number of plasma samples was 30 because 4 plasma samples were not collected.

b The number of urine sediment samples was 44 because 1 urine sediment sample was not collected.

c The number of plasma samples was 44 because 1 plasma sample was not collected.

d The number of urine sediment samples was 207 because 1 urine sediment sample was not collected.

e The number of plasma samples was 203 because 5 plasma samples were not collected.

f *P* < 0.05, compared to latent syphilis.

g *P* < 0.05, compared to symptomatic neurosyphilis.

h *P* < 0.05, compared to urine supernatant at the same stages.

**FIG 1:**
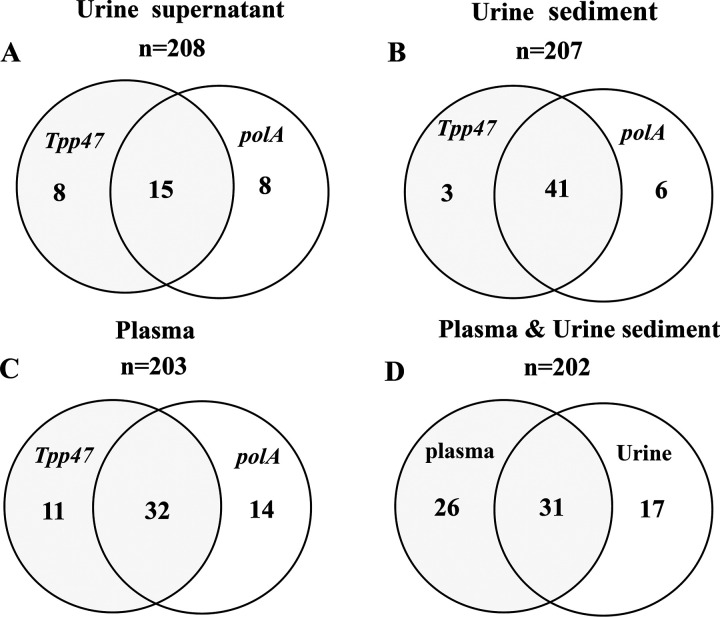
The detection of T. pallidum DNA in plasma and urine from all patients with syphilis by nested PCR (nPCR) assay. (A) Detection of *Tpp47* and *polA* in urine supernatant among all patients (*n* = 208). (B) Detection of *Tpp47* and *polA* in urine sediment (*n* = 207). (C) Detection of *Tpp47* and *polA* in plasma (*n* = 203). (D) Detection of *Tpp47* and *polA* in paired plasma and urine sediment (*n* = 202).

Among 31 positive urine supernatant samples, 15 were both *polA* and *Tpp47* positive (Fig. 1A), the κ coefficient being 0.61 (*P* < 0.0001) between *polA* and *Tpp47*. Among 50 positive urine sediment samples, 41 were both *polA* and *Tpp47* positive (κ = 0.87, *P* < 0.0001) (Fig. 1B). On the whole, 32 plasma samples were both *polA* and *Tpp47* positive, while 146 were both *polA* and *Tpp47* negative (κ = 0.64, *P* < 0.0001) (Fig. 1C). In 202 paired plasma and urine sediment samples, there were 31 concordance positive and 128 concordance negative in both plasma and urine sediment (Fig. 1D) (κ = 0.45, *P* < 0.0001). A total of 17 patients (5 primary, 7 secondary, 3 latent, and 2 symptomatic neurosyphilis) were T. pallidum DNA positive in urine sediment and negative in plasma, and there were 26 patients (7 primary,15 secondary, 2 latent, and 2 symptomatic neurosyphilis) who were T. pallidum DNA positive in plasma and negative in urine sediment.

### Primary and secondary syphilis and plasma T. pallidum DNA positivity were associated with the detection of T. pallidum DNA in urine.

We then analyzed the factors associated with the detection of T. pallidum DNA in urine by logistic regression. As shown in Table 3, compared with symptomatic neurosyphilis, primary and secondary syphilis were significantly associated with positive T. pallidum DNA in urine, with an adjusted odds ratio (OR) of 12.01 (1.61 to 89.7) (*P* = 0.015) and 10.64 (2.61 to 43.32) (*P* < 0.0001), respectively. Plasma T. pallidum DNA positivity was also associated with positive urine T. pallidum DNA with an adjusted OR of 2.05 (1.0 to 4.18) (*P* = 0.049).

**TABLE 3:** Univariate and multivariate analysis of the factors associated with T. pallidum DNA positive in urine from syphilis patients^*a*^

Variables	Univariate analysis			Multivariate analysis		
	Urine T. pallidum DNA positive (*n* = 52)	Urine T. pallidum DNA negative (*n* = 155)	*P* value	Adjusted OR	95% CI	*P* value
Age, median (IQR) (yr)	43 (30 to 56)	54 (34.25 to 61)	0.088	1.02	0.99 to 1.04	0.28
Male (%)	34 of 52 (65.4%)	99 of 155 (62.6%)	0.87			
1/serum RPR titer ≥ 32	40 of 52 (76.9%)	97 of 155 (62.6%)	0.062	1.88	0.71 to 4.99	0.21
Blood T. pallidum DNA positive	31 of 50 (62%)	26 of 152 (17.1%)	<0.0001	2.05	1.0 to 4.18	0.049
Periurethral lesions, *n* (%)^*b*^	21 of 53 (40.4%)	23 of 155 (14.8%)	<0.0001	1.08	0.26 to 4.47	0.91
HIV infection, *n* (%)	10 of 52 (19.2%)	11 of 154 (7.1%)	0.038	1.01	0.36 to 2.84	0.99
Stages						
Primary	16 of 52 (30.8%)	18 of 155 (11.6%)	<0.0001	12.01	1.61 to 89.7	0.015
Secondary	29 of 52 (55.8%)	32 of 155 (20.6%)	<0.0001	10.64	2.61 to 43.32	<0.0001
Latent	4 of 52 (7.7%)	64 of 155 (41.3%)	0.84	1.01	0.36 to 2.84	0.99
Symptomatic neurosyphilis	3 of 52 (5.8%)	41 of 155 (26.5%)	1	1		

*^a^*CI, confidence interval; IQR, interquartile range; OR, odds ratio; RPR, rapid plasma regain.

*^b^*“Periurethral lesions” refers to chancre and/or condyloma latum in the periurethral orifice.

### The quantity of T. pallidum DNA in urine and plasma.

To measure the loads of T. pallidum DNA in urine and plasma, 30 positive urine supernatant, 48 positive urine sediment, and 54 positive plasma samples were further quantified by ddPCR assay. As shown in Table 4, the loads of *polA* and *Tpp47* were 18.7 copies/mL (interquartile range [IQR], 0 to 80.85 copies/mL) versus 49.5 copies/mL (IQR, 18.7 to 153.5 copies/mL) in urine supernatant, 148.5 copies/mL (IQR, 4.95 to 649 copies/mL) versus 101.2 copies/mL (IQR, 19.8 to 1,029 copies/mL) in urine sediment, and 19.8 copies/mL (IQR, 0 to 37.95 copies/mL) versus 19.8 copies/mL (IQR, 0 to 44.0 copies/mL) in plasma, respectively. The loads of *polA* and *Tpp47* in urine sediment were significantly higher than those in plasma (both *P* < 0.05). The loads of *polA* in urine sediment were higher than those in urine supernatant (*P* < 0.05).

**TABLE 4:** The quantity of *polA* and *Tpp47* in urine and plasma in patients with different stages of syphilis^*a*^

Stages	Urine supernatant (copies/mL)		Urine sediment (copies/mL)		Plasma (copies/mL)	
	*polA*	*Tpp47*	*polA*	*Tpp47*	*polA*	*Tpp47*
Primary	59.4 (16.5 to 1,551)	50.6 (9.9 to 1,562)	148.5 (10.45 to 1,216)	59.4 (5.5 to 1,331)	23.1 (17.6 to 50.05)	27.5 (0 to 62.15)
Secondary	17.6 (0 to 20.35)	44 (11.55 to 66.55)	209 (22 to 649)^*b*^^,^^*c*^	253 (29.15 to 1,029)^*c*^	19.8 (0 to 37.4)	19.8 (0 to 43.45)
Latent	88 (0 to 176)	291.5 (55 to 528)	0 (0 to 198)	220 (0 to 220)	0 (0 to 0)	20.9 (0 to 41.8)
Symptomatic neurosyphilis	0	55	0	0	0 (0 to 0)	0 (0 to 0)
All cases	18.7 (0 to 80.85)	49.5 (18.7 to 153.5)	148.5 (4.95 to 649)^*b*^^,^^*c*^	101.2 (19.8 to 1,029)^*c*^	19.8 (0 to 37.95)	19.8 (0 to 44)^*b*^

*^a^*The loads of *polA* and *Tpp47* in urine supernatant from all cases (*n* = 30), primary syphilis (*n* = 9), secondary syphilis (*n* = 18), latent syphilis (*n* = 2), and symptomatic neurosyphilis (*n* = 1) are shown. The loads of *polA* and *Tpp47* in urine sediment from all cases (*n* = 48), primary syphilis (*n* = 16), secondary syphilis (*n* = 28), latent syphilis (*n* = 3), and symptomatic neurosyphilis (*n* = 1) are also shown. Finally, the loads of *polA* and *Tpp47* in plasma from all cases (*n* = 54), primary syphilis (*n* = 14), secondary syphilis (*n* = 36), latent syphilis (*n* = 2), and symptomatic neurosyphilis (*n* = 2) are shown.

*^b^P* < 0.05 compared to urine supernatant.

*^c^P* < 0.05 compared to plasma.

For primary syphilis, the loads of *polA* and *Tpp47* were 23.1 copies/mL (IQR, 17.6 to 50.05copies/mL) versus 27.5 copies/mL (IQR, 0 copy/mL-62.15copies/mL) in plasma, 148.5copies/mL (IQR, 10.45 to 1,216 copies/mL) versus 59.4copies/mL (IQR, 5.50 to 1,331 copies/mL) in urine sediment, and 59.4 copies/mL (IQR, 16.5to 1,551 copies/mL) versus 50.6 copies/mL (IQR, 9.9 to 1,562 copies/mL) in urine supernatant, but there was no significant difference among them. For secondary syphilis, *polA* and *Tpp47* reached 209 copies/mL (22 to 649 copies/mL) and 253 copies/mL (29.15 to 1,029 copies/mL), respectively, in urine sediment; these values are significantly higher than those in plasma (19.8 copies/mL [IQR, 0 to 37.4 copies/mL] versus 19.8 copies/mL [IQR, 0 to 43.45 copies/mL]) (*P* < 0.05 in both). The loads of T. pallidum DNA in urine and plasma were undetectable by ddPCR assay in patients with symptomatic neurosyphilis. In latent syphilis, only 1 sample among 3 positive urine sediment samples was detectable, and the loads of *polA* and *Tpp47* were 198 and 220 copies/mL, respectively. In 2 positive plasma samples, only 1 sample was detectable, and the loads of *Tpp47* were 41.8 copies/mL in latent syphilis.

## DISCUSSION

There is a growing interest in detecting a pathogen’s nucleic acid in various tissues in many infectious diseases (16, 17). The evidence showed that T. pallidum DNA could be detected by PCR assays in urine, especially in earlier syphilis patients (13–15). The study of Dubourg et al. showed that 4 patients were urine T. pallidum DNA positive among 25 syphilis patients (13). Gayet-Ageron et al. reported that the sensitivity of urine specimens was 29% (2 of 7) in primary syphilis patients and 44% (4 of 9) in secondary syphilis patients (14). In a recent study, 6% of patients were urine T. pallidum DNA positive among 200 men who had sexual intercourse with men (MSM) with early syphilis (15). However, to our knowledge, the detection of T. pallidum DNA in urine in all stages of syphilis has not been reported. To understand the T. pallidum DNA detection rates and loads in different urine components in syphilis patients at different stages, we tested early, latent, and even symptomatic neurosyphilis patients.

*polA* and *Tpp47* were chosen as target genes for detecting T. pallidum DNA in many previous studies (3, 13–15). *Tpp47* encodes a cytoplasmic-membrane protein involved in cell wall synthesis and is partially specific for T. pallidum subsp. *pallidum* (18). *polA* encodes DNA polymerase I involved in DNA repair and replication in most bacteria and contributed to a number of unique features in T. pallidum subsp. *pallidum* (19). The *Tpp47* and *polA* genes had good congruence in urine and plasma samples, the κ coefficients being more than 0.6 (*P* < 0.0001) in this study. Our results showed that 24.2% (50 of 207) syphilis patients had T. pallidum DNA detectable in their urine sediment, which was higher than that (14.9%) in urine supernatant, and 47.1% (16 of 34) primary syphilis and 47.5% (29 of 61) secondary syphilis cases were T. pallidum DNA positive in urine sediment. Our findings indicated that T. pallidum DNA could be present in the urine of syphilis patients at all stages, especially in early syphilis. Compared with previous studies, our results suggested that the amplification of T. pallidum DNA in urine sediment by nPCR assay could increase the detection rate. T. pallidum or DNA in urine could be concentrated by centrifugation. Centrifugation of urine samples that concentrated whole Borrelia organisms resulted in an improved detection rate (10). In addition, nPCR was more sensitive compared to single-step PCR, especially under the condition of small amounts of templates. However, many factors can affect DNA detection in urine by PCR assay, with urea being the major inhibitor among them (10, 20). As reported, the urea at a concentration greater than or equal to 50 Mm can inhibit *Taq* polymerase (21, 22), and the normal concentration of urea in adult urine is about 330 Mm. Therefore, the fact that the LOD of T. pallidum in plasma was lower than that in urine in our study indicated that optimizing PCR conditions were needed to increase the sensitivity.

We found that T. pallidum DNA can be detected in urine, but detection of short regions of T. pallidum by PCR cannot discriminate between DNA fragment and whole T. pallidum. A total of 41 samples showed *polA* and *Tpp47* coexistence among 50 positive urine sediment samples, which indicated good concordance between the two genes (κ = 0.87). The amplicons of *polA* and *Tpp47* were 376 and 354 bp in the first cycle amplification, respectively. If it was only DNA fragments, there should not be such good concordance. The study of Manion et al. showed viable B. burgdorferi in the urine of two clinically normal horses (23). Therefore, we speculated that it could be whole T. pallidum in urine. However, this remains to be further confirmed by culture or rabbit inoculation test.

T. pallidum DNA in urine could come from many sources. First, the lesions near the urethra in primary and some secondary syphilis patients could contaminate the urine. Second, T. pallidum can invade any organ, including the urinary tract and kidney (24–27). Latent or secondary syphilis-associated glomerulonephritis and nephrotic syndrome have been reported (23–28) as well. Thus, T. pallidum invading urinary systems could be excreted into the urine. Third, secondary syphilis, positive plasma T. pallidum DNA were significantly associated with the detection rate of T. pallidum DNA in urine sediment, and the adjusted ORs in this study were 10.64 and 2.05, respectively. The concordance of T. pallidum DNA between plasma and urine sediment (κ = 0.45) (*P* < 0.0001) indicated that blood T. pallidum or DNA could reach urine through the kidney barrier.

To our knowledge, this is the first report showing that T. pallidum DNA can be detected in urine at all stages of syphilis and that loads of T. pallidum DNA in urine sediment were significantly higher than those in plasma. Given the fact that urine is an excreta abundant in amount and that the collection of urine is noninvasive, convenient, and harmless to humans, urine is an attractive sample to acquire a certain amount of T. pallidum DNA. Therefore, urine could be an ideal supplement sample for further studies, such as detecting the molecular type of T. pallidum if no lesions are present.

## MATERIALS AND METHODS

### Ethics statement and subjects.

The study was approved by the Ethics Committee of the Shanghai Skin Disease Hospital. From March 2018 to January 2019, eligible syphilis patients who visited the Sexually Transmitted Disease (STD) Clinic of the Shanghai Skin Disease Hospital were invited to participate in this study. Patients were interviewed with a brief questionnaire to collect medical and social-demographic information. Patients who had treatment for syphilis prior to sample collection and who had a history of renal disease were excluded. In addition, 30 volunteers from the STD Department of the Shanghai Skin Disease Hospital were included in this study. Their serological tests for syphilis were negative, and there were no manifestations of syphilis. Written informed consent was obtained from all participants.

### Diagnostic criteria for primary, secondary, latent syphilis, and symptomatic neurosyphilis.

The diagnosis of syphilis is based on the guideline of the STD Association, China Centers for Disease Control (CDC) (29). Primary syphilis is defined as having clinical manifestation(s) of chancre(s) or ulcer(s) and at least having one of the following laboratory confirmations: (i) positive spirochetes by dark-field microscopic examination; (ii) positive serum RPR confirmed by TPPA; or (iii) negative spirochetes by dark-field microscopic examination and initial negative serum RPR and TPPA, but serum RPR and TPPA turned positive in the follow-up (maximum 3 months). Secondary syphilis is defined as (i) positive serum RPR confirmed by TPPA and (ii) skin or mucocutaneous lesions. Latent syphilis is defined as having positive serum RPR confirmed by TPPA, but without any lesions or symptoms of syphilis. Symptomatic neurosyphilis is confirmed by (i) positive serum RPR and TPPA; (ii) reactive cerebrospinal fluid (CSF) -venereal disease research laboratory test (VDRL) and CSF-TPPA or nonreactive CSF-VDRL but reactive CSF-TPPA with CSF protein concentration greater than 45 mg/dL and/or CSF white blood cell (WBC) counts greater than or equal to 8/μL; and (iii) clinical neurological or psychiatric manifestations without other known causes. Symptomatic neurosyphilis is classified into meningitis, meningovasculitis, general paresis, and tabes dorsalis according to clinical manifestations. Ocular neurosyphilis is defined as neurosyphilis patients with the presence of ocular signs or symptoms without other known causes.

### Samples collection.

Blood (5 mL) with anticoagulant EDTA was centrifuged (1,800 × *g*, 10 min), and plasma was collected. Urine (15 mL) was centrifuged (1,800 × *g*,10 min), and urine supernatant was collected. The urine sediment was then resuspended in 1 mL of urine supernatant. In addition, urine supernatant and sediment were collected from 20 syphilis patients after treatment (2 primary syphilis, 16 secondary syphilis, 1 latent syphilis, and 1 ocular neurosyphilis), whose urine was T. pallidum DNA positive before treatment. Samples from 30 volunteers were collected, including saliva, plasma, urine supernatant, and urine sediment. All samples were frozen at −20°C until they were examined.

### The sensitivity and specificity of *polA* and *Tpp47* detection in urine and plasma by nPCR assay.

To evaluate the sensitivity of *polA* and *Tpp47* detection in urine and plasma by nPCR assay, the LOD of *polA* and *Tpp47* was examined by extraction and amplification of experimentally spiked urine or plasma, in which T. pallidum (Nichols strain) was serially diluted to 3 × 10^4^, 3 × 10^3^, 3 × 10^2^, 3 × 50, 3 × 10, 3 × 5, 3, 0.3, and 0 T. pallidum/mL with plasma or urine from volunteers. To validate the specificity of *polA* and *Tpp47* by nPCR assay, *polA* and *Tpp47* were detected by extraction and amplification of control samples collected from saliva in 30 volunteers, plasma in 10, urine supernatant in 30, and urine sediment in 30. DNA extraction and amplification of *polA* and *Tpp47* were performed as described in our previous study (3). The PCR products were electrophoresed on a 2% agarose gel together with a 50-bp DNA ladder (TaKaRa) at 120 V for 30 min and visualized after staining with ethidium bromide.

### DNA extraction and PCR assays.

DNA was extracted from 1 mL urine supernatant, 1 mL urine sediment, and 1 mL plasma using the QiaAmp DNA blood minikit (Qiagen Inc., Germany) according to the manufacturer’s instructions. DNA was dissolved with 100 μL Tris-EDTA (TE) buffer. The presence and loads of *polA* and *Tpp47* genes were measured by nPCR and ddPCR assays, which were performed as described in previous studies (3, 4). For nPCR assay, there were 25 and 30 cycles in the first and second rounds of PCR, respectively. For ddPCR assay, there were 40 cycles.

### Statistical analysis.

We analyzed all data using SPSS software, version 24.0 (SPSS Inc., Chicago, IL). For categorical variables, we calculated proportions. For continuous variables, we calculated the median and interquartile range (IQR). We compared categorical variables, such as the detection rates of T. pallidum DNA by stages of syphilis and different samples, using the chi-square test or the Fisher’s exact test. We calculated the κ coefficients between the detection of T. pallidum DNA by different genes (*polA* and *Tpp47*) and different samples (plasma and urine) using standard formulae. The agreement of the results by κ value was categorized as almost perfect (0.81 to 1.0), substantial (0.61 to 0.80), moderate (0.41 to 0.60), fair (0.21 to 0.40), and slight (0.00 to 0.20). We used the Kruskal-Wallis H test (including Dunn-Bonferroni *post hoc* correction) to compare loads of T. pallidum DNA in urine supernatant, urine sediment, and plasma. Factors with a *P < *0.1 were included in the logistical regression. We considered *P < *0.05 with two-tailed tests to be statistically significant.
