# Supplementary material for: Quantified Detection of Treponema pallidum DNA by PCR Assays in Urine and Plasma of Syphilis Patients
Source: Microbiol Spectr. 2022 Mar 22;10(2):e01772-21. doi: 10.1128/spectrum.01772-21 (PMC9045283; doi:10.1128/spectrum.01772-21)
Supplement: SUPPLEMENTAL FILE 1 — Supplemental material. Download SPECTRUM01772-21_Supp_1_seq10.pdf, PDF file, 0.4 MB [file spectrum01772-21_supp_1_seq10.pdf]

Supplementary Figure 1

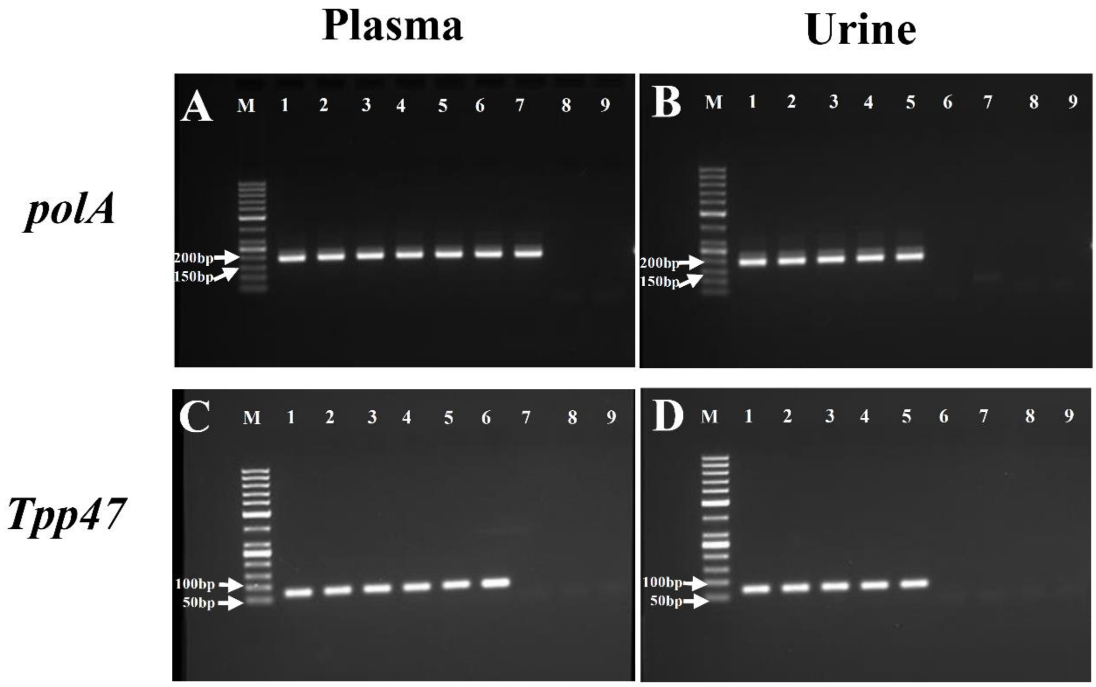

Supplementary Figure 1 legend

The limit of detection of *polA* and *Tpp47* in plasma and urine by nPCR assay.

Different number of *T. pallidum* (Nichols strains) were diluted in plasma (A) and urine (B) and *polA* gene was detected by nPCR assay; Different number of *T. pallidum* (Nichols strains) were diluted in plasma (C) and urine (D) and *Tpp47* gene was detected by nPCR assay. M: marker; line1, $3*10^4$ *T. pallidum*/mL; line 2, $3*10^3$ *T. pallidum*/mL; line 3, $3*10^2$ *T. pallidum*/mL; line 4, 3*50 *T. pallidum*/mL; line 5, 3*10 *T. pallidum*/mL; line 6, 3*5 *T. pallidum*/mL; line 7, 3 *T. pallidum*/mL; line 8, 0.3 *T. pallidum*/mL; line 9, negative control (plasma or urine without *T. pallidum*).
